# Nuclear and Mitochondrial DNA Analyses of Golden Eagles (*Aquila chrysaetos canadensis*) from Three Areas in Western North America; Initial Results and Conservation Implications

**DOI:** 10.1371/journal.pone.0164248

**Published:** 2016-10-26

**Authors:** Erica H. Craig, Jennifer R. Adams, Lisette P. Waits, Mark R. Fuller, Diana M. Whittington

**Affiliations:** 1 Aquila Environmental, Fairbanks, Alaska, United States of America; 2 Department of Fish and Wildlife Sciences, University of Idaho, Moscow, Idaho, United States of America; 3 Forest and Rangeland Ecosystem Science Center, US Geological Survey, Boise, Idaho, United States of America; 4 US Fish and Wildlife Service Headquarters, Falls Church, Virginia, United States of America; University of Regina, CANADA

## Abstract

Understanding the genetics of a population is a critical component of developing conservation strategies. We used archived tissue samples from golden eagles (*Aquila chrysaetos canadensis*) in three geographic regions of western North America to conduct a preliminary study of the genetics of the North American subspecies, and to provide data for United States Fish and Wildlife Service (USFWS) decision-making for golden eagle management. We used a combination of mitochondrial DNA (mtDNA) D-loop sequences and 16 nuclear DNA (nDNA) microsatellite loci to investigate the extent of gene flow among our sampling areas in Idaho, California and Alaska and to determine if we could distinguish birds from the different geographic regions based on their genetic profiles. Our results indicate high genetic diversity, low genetic structure and high connectivity. Nuclear DNA Fst values between Idaho and California were low but significantly different from zero (0.026). Bayesian clustering methods indicated a single population, and we were unable to distinguish summer breeding residents from different regions. Results of the mtDNA AMOVA showed that most of the haplotype variation (97%) was within the geographic populations while 3% variation was partitioned among them. One haplotype was common to all three areas. One region-specific haplotype was detected in California and one in Idaho, but additional sampling is required to determine if these haplotypes are unique to those geographic areas or a sampling artifact. We discuss potential sources of the high gene flow for this species including natal and breeding dispersal, floaters, and changes in migratory behavior as a result of environmental factors such as climate change and habitat alteration. Our preliminary findings can help inform the USFWS in development of golden eagle management strategies and provide a basis for additional research into the complex dynamics of the North American subspecies.

## Introduction

An understanding of genetic diversity and structure is vital for developing conservation strategies for species of concern [1]. Generally, contemporary species management strives to conserve genetic diversity, which is especially important in times of accelerated environmental change [2]. Genetic analyses can identify metapopulations, subpopulations, source and sink populations, and investigate migratory connectivity, changes in phylogeographic patterns in response to climate change and other biological or evolutionary differences among population segments [1,3–11]. Genetic analyses of North American (NA) raptors have generally shown high levels of contemporary gene flow and a low degree of genetic structure across large spatial scales [7,12,13], but recent research by Doyle et al. [14] using 162 single nucleotide polymorphisms (SNPs) at neutral and adaptive loci indicate that golden eagles in NA may exhibit some structure across their range.

Golden eagles (*Aquila chrysaetos*) are Holarctic in distribution and some studies have evaluated genetic diversity and structure for the species [11,15–18]. These studies have generally found high levels of diversity and connectivity. However, in NA, where only one subspecies of golden eagle (*A*. *c*. *canadensis*) occurs, Katzner et al. [18] report that eagles from east of the Mississippi River exhibited some degree of geographic isolation from those westward prior to reintroductions, and historically might have been genetically distinct. More recently, Doyle et al. [14] found pronounced genetic structure among golden eagles from four geographic sampling sites in NA.

Status of the golden eagle population in the western contiguous US is uncertain; count data suggest a stable population [19] but more recent demographic models and satellite-tag data predict a slight decline [20]. Katzner et al. [18] concluded that golden eagles in eastern NA are increasingly at risk from threats on wintering, migration and breeding grounds. At localized scales, nest occupancy and counts of birds in parts of the western US also show evidence of declines [19,21,22] and increasing risks within their range. The status of northern latitude eagles is uncertain, but Kochert et al. [23], using data largely from Denali National Park, Alaska, suggested population stability in Alaska. Recently, McIntyre and Schmidt [24] reported that long-term occupancy of nests in Denali National Park remains stable, but that breeding performance is declining. Currently, the rapid expansion of energy development [25] in the contiguous US is a concern for population status [26,27], in part because carcasses of golden eagles are routinely found at some wind energy projects [28,29].

The USFWS, under the Bald and Golden Eagle Protection Act (Eagle Act, 16 U.S.C. 668-668d), has primary authority for the management of golden eagles in the US. Some golden eagles are harvested by Native Americans and falconers (see Code of Federal Register 50 CFR 22.22–22.24); current management also allows for additional “take” of golden eagles under certain circumstances. The Eagle Act defines “take” as “…pursue, shoot, shoot at, poison, wound, kill, capture, trap, collect, or molest or disturb…”. Recently, the USFWS began to permit a limited unintentional “take” of eagles at energy projects [30], but within the context of maintaining a stable or increasing eagle population over 100 years [31]. Characteristics of golden eagle behavior can complicate design and implementation of management, including monitoring population status. NA golden eagles exhibit a variety of migration strategies. Segments of the population undergo long annual migrations from Alaska and northern Canada [32,33], others migrate shorter distances, and some remain as year-round residents in their nesting areas [23]. They exhibit dispersal movements and responses to resources that result in individuals using disparate locales during the course of their life cycle and mixing with birds from other geographic natal areas [23]. As a result, the NA population is exposed to factors across a broad geographic scale [23] that have the potential for negative cumulative effects on eagle population status. The consequences of permitted take or other factors that influence survival or fitness in one locale or management unit might affect individuals that disperse or migrate elsewhere, making it difficult to assess the effects of management. However, there is a paucity of data about dispersal and migration movements to inform managers about the potential risks from localized perturbations (e.g., wind energy production) for birds in other areas of the subpopulation or to the population as a whole.

Genetic data can help identify subpopulations and are useful for designing conservation and management plans [2,13,15,34,35]. Genetic information is especially relevant for current considerations by USFWS: 1) of natal dispersal and movements, 2) for establishing the size and location of eagle management units (EMU), 3) for setting an upper limit on eagle take at a smaller scale than the EMU, to avoid creating population sinks in local breeding populations, 4) for evaluating the extent of geographic area potentially affected by permitting of anthropogenic related fatalities, 5) for evaluating the effects and locations of mitigation associated with permits, and 6) for monitoring the outcome of management [31].

We investigated population genetic variation and structure among sampled golden eagles from three geographic areas in western NA using a combination of mitochondrial DNA (mtDNA) D-loop sequences and microsatellite loci. We used archived blood samples from birds that spent the summer (breeding) season in Idaho/eastern Oregon, in California, and in Alaska and samples of eagles wintering in Idaho that represented both migrants and year-round residents. Our research goals were to: 1) investigate the extent of gene flow among golden eagles from these three geographic areas, 2) determine if summer resident eagles from different geographic locations in western NA can be distinguished based on their genetic profiles and 3) determine if the natal origin of winter residents in Idaho can be determined from their genetic profiles. We discuss our results as initial data for understanding the genetics of the subspecies, as a consideration for USFWS in evaluating the effects of permitting actions, and in development of conservation and management plans that must account for complex lifetime movements of individuals.

## Materials and Methods

### DNA Extraction

The samples from golden eagles were collected by the authors or were contributed (Table A in S1 File). Field collection of tissue samples was conducted under federal permits issued by the US Geological Survey (USGS) Bird Banding Laboratory (BBL; Laurel, MD; range of collection dates: 1999–2011). Samples were from three geographic populations where golden eagles occur year-round: 1) south and central Idaho (n = 19) and adjacent eastern Oregon (n = 6; we refer to these 25 as Idaho samples in the text); 2) central California (n = 26), and 3) northern Alaska (n = 7). We refer to the samples from eagles known to occur in each geographic region during the summer (breeding season) as summer residents throughout the text. We defined summer as that period after migrants arrive during the breeding season until they migrate (31 March– 30 September). The Alaskan birds in our sample represent birds that summer in Alaska but migrate south for winter. We do not know the breeding status of every individual in our dataset. Samples from summer residents included: blood (*n* = 45), tissue (*n* = 3), feathers (*n* = 9) and fecal material (*n* = 1). In addition, we used blood samples from eagles that wintered in Idaho (*n* = 9) to determine if we could assign them to geographic summer locations based on our results from known origin birds. DNA was extracted from blood, tissue and feather samples using a modified DNeasy Blood and Tissue Kit protocol (Qiagen, Inc. Germantown, Maryland, USA) [36,37], and from the fecal sample using the QIAmp DNA Stool Mini Kit protocol (Qiagen, Inc). DNA extraction from the feathers and fecal sample occurred in a laboratory dedicated to low quality DNA samples with no forms of concentrated golden eagle DNA present. An extraction negative was included in each extraction to test for reagent contamination.

### Microsatellite Genotyping

Thirty-six microsatellite loci, provided by Deborah Dawson (Natural Environment Research Council, Biomolecular Analysis Facility, Sheffield, England), were tested for amplification success and variability in birds from Idaho (*n* = 6) and California (*n* = 6; Table B in S1 File). Eighteen loci were chosen for further analysis based upon the number of alleles, the observed and expected heterozygosity, and the ability to multiplex the loci into two polymerase chain reactions (PCRs; Table B in S1 File).

Multiplex PCR 1 contained 1X Qiagen Multiplex Master Mix, 0.5X Q-solution 0.20 μM of Hal10, 0.15μM of IEAAAG15, 0.12 μM of Aa35, 0.10 μM of Aa11, Aa36, and IEAAAG13, 0.09 μM of Aa02 and Aa27, 0.07 μM of Hal09 and Hal13, 0.06 μM of IEAAAG14 and 0.05 μM of Aa04, Aa26, and NVHfr142, and 1.0 μl of DNA extract in a 10 μl reaction volume. Multiplex PCR 2 contained 1X Qiagen Multiplex Master Mix, 0.5X Q-solution, 0.10 μM of Aa49, 0.13 μM of Aa39, 0.14 μM of BV13, 0.29 μM of Aa43 and 1.0 μl of DNA extract in a 7 μl reaction volume. The thermocycler profile for Multiplex PCR 1 was an initial denaturation step of 94°C for 15 min followed by 10 cycles of 94°C for 30 sec, touchdown 62°C—57°C for 90 sec, and 72°C for 1 min, followed by an additional 30 cycles of an annealing temperature of 57°C with a final extension of 60°C for 30 min. The thermocycler profile for Multiplex PCR 2 was the same as above except the touchdown was 62°C—54°C and 30 cycles of an annealing temperature of 54°C. All PCRs were run with a negative control to test for reagent contamination. PCR products were run on an Applied Biosystems 3130xl Genetic Analyzer and scored using GeneMapper v3.7 (Applied Biosystems, Inc, Foster City, CA USA).

### Mitochondrial DNA Sequencing

The sample of western golden eagles was also assessed using mitochondrial DNA (mtDNA) sequencing of the D-loop region (also known as the control region). A ~415bp fragment of the D-loop region was amplified using the primers GOEA_CR1L and GOEA_CR595H [17]. PCR products were prepared for sequencing using ExoSAPit according to the manufacturer’s protocol (USB; Affymetrix, Santa Clara, CA USA). Sequencing was performed using a quarter reaction of the BigDye^®^ Terminator v3.1 Cycle Sequencing Kit. Sequencing products were cleaned using the BigDye^®^ Xterminator^™^ Purification Kit according to the manufacturer’s protocol (Applied Biosystems, Inc.). Sequences were run on a 3130xl Genetic Analyzer (Applied Biosystems, Inc.) and analyzed using Sequencher 4.7 (Gene Codes Corporation, Ann Arbor, MI USA). Sequences were aligned by eye in Sequencher 4.7 (Gene Codes Corporation), and the number of haplotypes was determined using the filter redundant taxa option in MacClade 4.08 [38]. Golden eagle haplotypes from other studies in North America [9,17] were downloaded from Genbank (www.ncbi.nlm.nih.gov/genbank/).

### Genetic diversity estimates

The number of alleles, observed and expected heterozygosity and the probability of identity (PID and PIDsibs) were calculated for each microsatellite locus using Gimlet 1.3 [39]. The eighteen loci were tested for Hardy-Weinberg equilibrium (HWE) using Genepop 4.0 [40]. Private alleles were identified in each population using GenAlEx 6.4 [41]. Haplotype and nucleotide diversity were calculated for the mtDNA D-loop haplotype data using Arlequin 3.5 [42]. Tests of selective neutrality (Tajima’s D [43] and Fu’s F_s_ [44]) were performed for the California and Idaho populations in Arlequin 3.5 [42].

A median joining network was drawn using Network 5 (Fluxus Technology Ltd., Clare, Suffolk UK). Included in the network were sequences from an additional 6 eagles that summered in Alaska (provided by Robert Domenech, Raptor View Research Institute, Missoula, MT USA; Peter Sherrington, Rocky Mountain Eagle Research Foundation, Calgary, AB Canada and genetic analysis done by Sean Rogers, University of Calgary, Calgary, AB Canada).

### Population differentiation

The samples from Alaska were excluded from population differentiation analyses due to small sample size. The degree of genetic differentiation between California and Idaho summer resident golden eagles was assessed by calculating pairwise F_ST_ in Arlequin 3.5 for both the microsatellite loci and the D-loop haplotype data [42,45].

To evaluate whether individuals can be classified into genetic groups based on their genetic signature, we used an assignment test approach as implemented in the software Geneclass 2 [46,47], plus the Bayesian clustering methods implemented in the programs STRUCTURE 2.3 [48,49] and BAPS 5.4 [50]. In STRUCTURE we determined the most likely number of populations (K) by identifying the value of K with the lowest log-likelihood. The value of K was varied from 1–5 and 10 replicates were performed for each value of K. All STRUCTURE runs used the admixed ancestry model and the correlated allele frequencies model with a burn-in length of 100,000 repetitions followed by 400,000 MCMC iterations. We also estimated the most likely value of K (1–5) using the aspatial model in BAPS 5.4 and 10 replicates were performed for each value of K. An Analysis of Molecular Variance (AMOVA; [51]) was performed on the mtDNA D-loop haplotype data to determine the proportion of haplotype variation that could be attributed to the population level using the program Arlequin 3.5.

## Results

### Genetic Diversity

Twenty-five of the 36 microsatellite loci amplified successfully and produced bands in the correct size ranges (Table B in S1 File). Although eighteen loci were chosen for analysis, the final dataset only includes 16 loci because of frequent amplification failure at loci Aa02 and Aa43. We obtained genotype data for 54 of the 58 individuals (Idaho = 24, California = 24, Alaska = 6). Two blood samples, one feather sample, and one fecal sample failed to produce enough DNA for analysis. The average observed and expected heterozygosities per locus were similar across the three populations (Table 1). The total number of alleles per locus ranged from 2–14 and the average PID and PIDsibs per locus was 0.249 and 0.535 respectively (Table B in S1 File). Diversity calculations given in Table B in S1 File for Aa02 and Aa43 were obtained from a smaller dataset (Aa02 n = 27, Aa43 n = 15). We removed these loci from all further analyses. Two of 16 loci from our Idaho samples (Aa49 and Hal09) and two from California (Aa36 and Hal09) were not in Hardy-Weinberg equilibrium using p ≤ 0.05 as a threshold. With the Bonferroni corrected p-value for multiple tests (p < 0.0031), all loci in the Idaho and California populations were in Hardy-Weinberg equilibrium. The Idaho summer residents had 14 private alleles at 9 loci, the California eagles had 17 private alleles at 10 loci and the Alaska eagles had 1 private allele at one locus (Table 1).

**Table 1:** The number of alleles (N_a_), expected (H_e_) and observed (H_o_) heterozygosities and number of private alleles (PA) for 16 nuclear DNA microsatellite loci summarized for each golden eagle population.

	Alaska (n = 6)				California (n = 24)				Idaho (n = 24)			
Locus	N_a_	H_e_	H_o_	PA	N_a_	H_e_	H_o_	PA	N_a_	H_e_	H_o_	PA
Aa04	6	0.74	0.50	1	11	0.82	0.79	4	9	0.75	0.71	1
Aa11	5	0.74	0.67		5	0.74	0.79	1	5	0.73	0.83	
Aa26	3	0.29	0.33		6	0.53	0.54	2	4	0.57	0.54	
Aa27	4	0.42	0.50		5	0.62	0.58		4	0.41	0.33	
Aa35	2	0.28	0.33		3	0.22	0.25	1	2	0.33	0.25	
Aa36	4	0.68	0.33		6	0.61	0.63		7	0.63	0.58	2
Aa39	4	0.63	0.67		7	0.57	0.58	2	5	0.56	0.63	
Aa49	3	0.54	0.83		7	0.78	0.68	2	6	0.68	0.92	1
BV13	4	0.64	0.83		5	0.50	0.53	1	5	0.60	0.68	1
Hal09	3	0.49	0.67		6	0.77	0.46		8	0.74	0.75	3
Hal10	4	0.60	0.67		5	0.67	0.83		7	0.76	0.70	2
Hal13	2	0.38	0.50		2	0.12	0.13		2	0.44	0.42	
IEAAAG13	2	0.28	0.33		2	0.28	0.25		2	0.22	0.25	
IEAAAG14	2	0.49	0.17		3	0.57	0.71		3	0.47	0.54	
IEAAAG15	3	0.57	1.00		3	0.48	0.50		4	0.60	0.54	1
NVHfr142	1	0	0		4	0.47	0.50		4	0.30	0.33	
Average	3.25	0.48	0.52		5	0.55	0.55		4.81	0.55	0.56	

**Fig 1:**
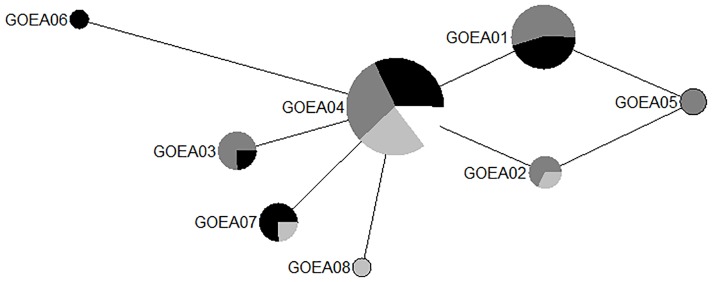
Parsimony network of eight D-loop mitochondrial DNA haplotypes for 60 golden eagle summer resident samples (Idaho = 21, California = 23, Alaska = 11, Canada = 5) from this study and others [9,17]. Node size is equivalent to the number of individuals with each haplotype. Each line is equivalent to one base pair change between haplotypes with the exception of the line between GOEA04 and GOEA06, which is equivalent to two base pair changes. Light gray = Alaska, dark gray = California, black = Idaho and white = Canada.

Sequence data were obtained at 409 base pairs of the D-loop region of mitochondrial DNA for 49 summer resident samples (Idaho = 21, California = 23, Alaska = 5) and all 6 samples from Alaska provided by Domenech and Sherrington. Eight mtDNA D-loop haplotypes were detected (GenBank accession numbers: KX687705—KX687711 for GOEA01—GOEA07, respectively; and JQ246418 for GOEA08, Table 2, Fig 1). The haplotypes were distinguished based on 7 variable sites (Table 2). Haplotypes GOEA03, GOEA06 and GOEA07 were unique to this study (Table 2, Fig 1). Four of the five haplotypes detected by Sonsthagen et al. [17] for California eagles, matched haplotypes found in our dataset (Table 2). Three mtDNA haplotypes were identified in the dataset from the 6 Alaskan eagles provided by Domenech and Sherrington; all three matched haplotypes identified in Sonsthagen et al. [17] and two matched haplotypes from our dataset ([17]; Table 2). The haplotype identified in 5 Canadian eagles by Nebel et al. [9] matched haplotype GOEA04 (Fig 1). Given our current sampling, haplotype GOEA05 appears to be specific to California while haplotype GOEA06 appears specific to Idaho (Table 2; Fig 1).

**Table 2:** Geographic locations (Alaska = AK, California = CA, Idaho = ID) where each of 8 mitochondrial D-loop region haplotypes were identified. The nucleotide variation of the 8 haplotypes at each of 7 variable sites is also listed.

								Location in base pairs						
Haplotype	AK	CA	ID	Domenech & SherringtonAK	Nebel et al. [9] Canada	Total	Sonsthagen et al. [17]	21	54	138	148	150	153	320
**GOEA01**		6	5			11	JQ246421	T	G	G	C	T	G	G
**GOEA02**		2		1		3	JQ246420	-	-	A	T	-	-	-
**GOEA03**		3	1			4		-	A	-	T	-	-	-
**GOEA04**	4	10	11	4	5	34	JQ246417	-	-	-	T	-	-	-
**GOEA05**		2				2	JQ246419	-	-	A	-	-	-	-
**GOEA06**			1			1		C	-	-	T	-	-	A
**GOEA07**	1		3			4		-	-	-	T	-	A	-
**GOEA08**				1		1	JQ246418	-	-	-	T	C	-	-

Haplotype (h) and nucleotide (π) diversity was similar between California (h = 0.74 ± 0.064 SD), (π = 0.0025 ± 0.0019 SD) and Idaho (h = 0.68 ± 0.085 SD), (π = 0.0023 ± 0.0018 SD) and lower for Alaska (h = 0.49 ± 0.18 SD), (π = 0.0013 ± 0.0013 SD). Tajima’s D and Fu’s F_S_ were not significantly different than zero in the California (D = 0.62, p = 0.77 and F_S_ = -0.91, p = 0.25) and Idaho populations (D = -1.01, p = 0.19 and FS = -1.26, p = 0.14).

### Population differentiation

The pairwise F_ST_ value calculated from the microsatellite data between Idaho and California was low but significantly greater than zero (0.026, p = 0.001) indicating that the two populations are not in panmixia, but genetic differentiation is low between the Idaho and California golden eagle populations. The pairwise F_ST_ value calculated from the D-loop haplotype data between Idaho and California was also low and not significantly greater than zero (0.030, p = 0.143).

**Fig 2:**
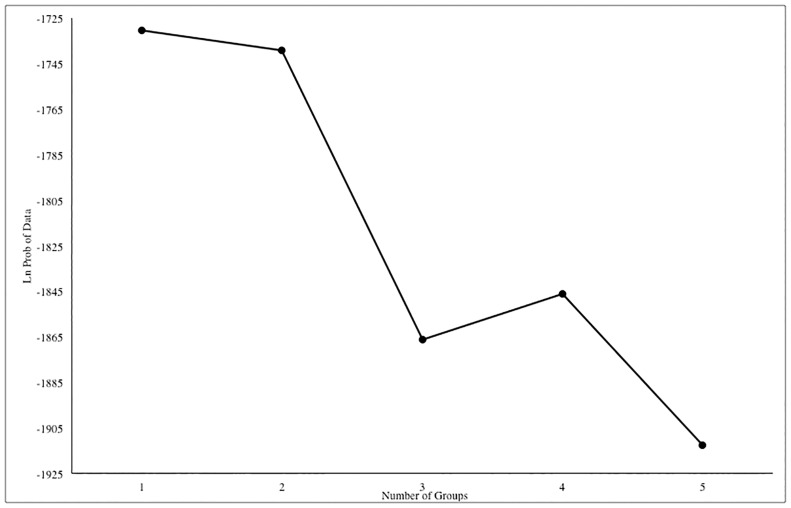
Program STRUCTURE log likelihood values for each value of K (number of genetic groups) using 16 loci of microsatellite data for golden eagles from Idaho (n = 24) and California (n = 24).

The assignment test implemented in Geneclass 2 correctly assigned an individual eagle to its sampling region of origin 60% of the time. This suggests there is not enough of a difference between allele frequencies in the California and Idaho populations to confidently assign individuals to their sampling population with the current number of loci. The results from program STRUCTURE indicate that the value of K with the greatest log likelihood is one when analyzing the Idaho and California samples only, and we obtained the same result when the 6 samples from Alaska were included (Fig 2). Program BAPS found the most likely number of groups to be five; this program is more likely to overestimate the number of groups with F_ST_ values less than 0.03 [52]. Three of the groups had only one or two individuals and were therefore not considered to be true groups. The remaining two groups did not clearly cluster along geographic boundaries.

Results of the AMOVA showed that 97% of the mtDNA haplotype variation was within the geographic populations while 3% of the haplotype variation was among populations (Table 3). Using genotypes from 16 microsatellite loci and 409 bases of the mitochondrial DNA D-loop region, we were unable to definitively differentiate golden eagle summer residents from three geographic locations or assign wintering birds to locales based on their genetic profiles.

**Table 3:** Results of the mtDNA AMOVA in the California and Idaho golden eagle populations.

Source of variation	Degrees of Freedom	Sum of squares	Variance components	Percentage of variation
**Among Populations**	1	0.81	0.015	3.0
**Within Populations**	42	20.37	0.486	97.0
**Total**	43	21.18	0.50	100.0

## Discussion

### Genetic findings

Similar to other work on raptors in NA [7,12,13], we found low levels of genetic structure and a high degree of genetic connectivity across our sampling distribution of golden eagles. Frequency-based assignment tests and Bayesian clustering methods could not definitively distinguish eagles from different geographic regions. Our mtDNA results also showed very little variation (3%) partitioned among populations and one common haplotype shared across Idaho, California and Alaska. However, two region-specific haplotypes were detected, one in the California and one in the Idaho samples; these may be unique to those areas. Additional sampling is needed to verify whether these are truly private haplotypes or just an artifact of limited sampling. In a recent genetic analysis of golden eagles in NA, Doyle et al. [14] also found 3% variation partitioned among their four geographic sampling sites (California, Alaska, western states, eastern states) and 97% within those sites. Their preliminary results provided evidence for significant genetic differentiation between California and other western states (Arizona, Colorado, Nebraska, New Mexico, Utah, Wyoming). Similarly, our findings of a significant deviation from panmixia at nDNA Fst and private alleles at multiple microsatellite loci suggest that gene flow is restricted to some degree between California and Idaho. Thus, it may be possible to distinguish eagles from different breeding grounds by analyzing additional loci as was demonstrated in Doyle et al. [14]. The recent completion of a genome sequence for golden eagles [53] and the identification of neutral and adaptive SNPs [14] will facilitate future analyses.

Our findings of low genetic structure and high connectivity are consistent with earlier preliminary comparisons of mtDNA and nDNA structure of 44 eastern and 25 western NA golden eagles that found no evidence for population substructure and shared mtDNA haplotypes across these two regions [18]. However, multiple reintroductions of eagles from the western populations to the Eastern US may have altered allele frequency distributions and artificially elevated connectivity in the sampled populations [18]. Distance and natural geographic barriers can reduce gene flow in populations [14]. However, recent experimental evidence indicates that the degree of connectivity in populations of migratory animals can increase as a result of habitat loss, and that geographic populations at sites not directly connected to the habitat loss can be affected [54]. The development of wind and solar energy projects across the western US is altering vast tracts of land [25] that include golden eagle winter and summer range. Poessel et al. [55] observed home range size of golden eagles to increase in fragmented urban landscapes. It is possible that habitat fragmentation could be one of the contributing factors to the connectivity we observed among the three sampling areas. However, results from Tajima’s D and Fu’s F_S_ using mtDNA sequence data from eagles in Idaho and California provide no evidence for recent population expansion or bottlenecks.

In contrast to our findings, Sonsthagen et al. [17] were able to distinguish genetic groups of golden eagles from the Channel Islands and the California mainland using 9 microsatellite loci and Bayesian clustering methods, but F_ST_ levels (0.03) also indicated high gene flow. Our study adds to the growing understanding of golden eagle genetics in NA and similar to Doyle et al.’s recent work [14], further demonstrates the need for increased sampling across the eagle range in NA, as well as, increasing the number of genetic markers.

### Implications for conservation and management

Genetic research can contribute to understanding the extent to which animals rely on resources and face threats beyond their natal area [54]. Some dispersing and migrating golden eagles will be affected by eagle management at locales such as wind energy project sites and conservation areas, but the effects of management could be manifested elsewhere. For example, mortality of eagles at a project site could contribute to a decline in number of birds at destination nest or winter sites and mistakenly be attributed to factors at the destination site. In order to manage populations for long term sustainability, it is vital to identify the geographic origin of individuals in a population that are killed or incur other negative demographic affects [35,56]. We were unable to determine origins of the wintering golden eagles in our sample. However, population genetic research has revealed relationships among individuals of other widely distributed populations of NA raptors that also exhibit a range of migration strategies [7,12,13]. Further genetics research, including the use of additional loci (e.g., [14]), will help clarify those relationships for golden eagles.

Natal dispersal is integral to genetic structure, species demography, and conservation [57]. In Idaho, some eagles nest 300 km or more from their natal areas [58], and banding and radio tracking data from NA reveal natal dispersals of 200–500 km [23,33,59]. Despite these long dispersals, the median natal dispersal distance based on banding data alone, is 46.4 km for golden eagles (n = 96; [59]). Millsap et al. [59] noted that the USFWS uses this estimated natal dispersal distance to set one of the geographic scales (46–175 km) for evaluating the effects of permits that allow take of golden eagles. Based on our results, genetic exchange is occurring at a scale larger than currently used by the USFWS, thus providing additional information for evaluating the geographic extent of the potential effects of eagle take from local areas and for deciding on the size and distribution of EMUs [27,31,34,60,61].

Breeding dispersal by adults also could contribute to gene flow among our sample populations. Unsuccessful golden eagle breeders can disperse [62] and reproduce elsewhere if conditions are more favorable, thus contributing to gene flow. Although breeding dispersal is poorly studied in golden eagles, and thought to be uncommon [23], non-territorial, non-breeding adults, termed floaters, are an important component of a raptor population [63,64]. Floaters are potential breeders and their existence implies that opportunities for breeding are limited (e.g., lack of nest sites [65]); this may result in floater dispersal to find suitable sites. A population of long-lived birds [like golden eagles] with few floaters, is vulnerable to decline [66]. When considering the possibility to allow take, the USFWS [30] used Moffat’s equilibrium [65] and the Millsap and Allen [67] analysis of anthropogenic demographic removal, and concluded the floating population of golden eagles in NA could be limited. Floaters likely affect genetic structure among geographic subpopulations and are important for sustaining or increasing the golden eagle population, especially in light of natural population fluctuations (e.g., [68]), managed take, and disturbances.

Understanding the role that floaters and other immigrants play in the population dynamics at a locale is difficult to do without knowledge of the genetics of that portion of the population. Undetected replacement of breeders can mask mortality in breeding locales [65]. For example, golden eagles in the Altamont Pass Wind Resource Area (APWR), California incurred high yearly mortality (67 eagles; 80% CI = 25–109) due to wind turbine blade strikes [28]. Most eagles in and around the APWR appeared to be resident, but in spite of high mortality, the number of nesting season pairs remained stable ([69]; G. Hunt and T. Hunt, pers. comm.). However, recent genetic and isotopes analyses of eagles killed in the APWR suggest this nesting season population may be sustained by immigration [70]. Including immigrants in breeding season surveys could indicate a stable breeding population, but mask high incidence of local mortality for nesting birds and fail to reveal source—sink population dynamics [71,72]. Further, simply monitoring mortality counts of eagles at wind generation project sites provides insufficient information about long-term effects on the spatial dynamics of eagle abundance. The addition of genetic data can contribute to detecting source—sink scenarios [73,74] and genetic monitoring can aid in the understanding of the genetic effects of harvest [35] on golden eagle subpopulations.

Golden eagles can encounter multiple risks (e.g., [75]) across their geographic range during annual cycles. Genetic assessment in combination with demography and movement ecology (e.g., [33,76]), and other methods [6,61,77,78] can inform adaptive management to accommodate the spatial scales of golden eagle movement and gene flow, and the temporal changes that might occur in response to take, mitigation measures [35], persecution [11,79] and other disturbances [78,80,81]. The basis of USFWS guidance for golden eagle management and for issuing permits to take eagles includes assessing cumulative effects in the “reasonably foreseeable future” [30] and at geographic scales from the project development site through the national scale [27]. Without supporting genetics it will be difficult to predict the potential cumulative effects of take [35] and other factors on golden eagle population status. Similar to Doyle et al. [14], we recommend extensive field sampling to assess genetic structure at the scale of eagle management units. The ecology and management of NA golden eagles [27,31] will benefit from building on recent genetic results [14,17] and the new genome sequence [53], and by continuing to use archived tissue samples, gathering and analyzing additional localized, seasonal samples, and by genetic monitoring [35,82].

## Supporting Information

S1 File Additional information for the golden eagle tissue samples used in the genetic analyses, and for each microsatellite locus that amplified in 54 golden eagles. (DOCX)
