# Supplementary material for: Nuclear and Mitochondrial DNA Analyses of Golden Eagles (Aquila chrysaetos canadensis) from Three Areas in Western North America; Initial Results and Conservation Implications
Source: PLoS One. 2016 Oct 26;11(10):e0164248. doi: 10.1371/journal.pone.0164248 (PMC5082654; doi:10.1371/journal.pone.0164248)
Supplement: S1 File — (DOCX) [file pone.0164248.s001.docx]

**Supporting Information**

**S1. Additional information for the golden eagle tissue samples used in the genetic analyses, and for each microsatellite locus that amplified in 54 golden eagles.**

**Table A. Contributors of golden eagle tissue samples used in the genetic analyses and additional sample information. Terminology for age is after Howell** [1] **and as listed by the sample contributor.**

| **Sample** | **State^1^** | **Contributor** | **Age** | **Tissue** | **Year Sampled** | **Summer Resident** |
| --- | --- | --- | --- | --- | --- | --- |
| **GECA01** | CA | Grainger Hunt, The Peregrine Fund, Boise, ID USA | Juvenile | blood | 1999 | yes |
| **GECA02** | CA | Grainger Hunt, The Peregrine Fund, Boise, ID USA | Subadult | blood | 1999 | yes |
| **GECA03** | CA | Grainger Hunt, The Peregrine Fund, Boise, ID USA | Subadult^2^ | blood | 2000 | yes |
| **GECA04** | CA | Grainger Hunt, The Peregrine Fund, Boise, ID USA | Juvenile | blood | 1999 | yes |
| **GECA05** | CA | Grainger Hunt, The Peregrine Fund, Boise, ID USA | Juvenile | blood | 1999 | yes |
| **GECA06** | CA | Grainger Hunt, The Peregrine Fund, Boise, ID USA | Subadult | blood | 1999 | yes |
| **GECA07** | CA | Grainger Hunt, The Peregrine Fund, Boise, ID USA | Subadult | blood | 1999 | yes |
| **GECA08** | CA | Grainger Hunt, The Peregrine Fund, Boise, ID USA | Subadult^2^ | blood | 1999 | yes |
| **GECA09** | CA | Grainger Hunt, The Peregrine Fund, Boise, ID USA | Hatch Year^4^ | blood | 1999 | yes |
| **GECA10** | CA | Grainger Hunt, The Peregrine Fund, Boise, ID USA | Adult^3^ | blood | 2000 | yes |
| **GECA11** | CA | Grainger Hunt, The Peregrine Fund, Boise, ID USA | Juvenile | blood | 1999 | yes |
| **GECA12** | CA | Grainger Hunt, The Peregrine Fund, Boise, ID USA | Near-adult | blood | 1999 | yes |
| **GECA13** | CA | Grainger Hunt, The Peregrine Fund, Boise, ID USA | Juvenile | blood | 1999 | yes |
| **GECA14** | CA | Terra Kelly, Christine Kreuder Johnson, University of California, Davis, CA USA | Adult | blood | 2007 | yes |
| **GECA15** | CA | Terra Kelly, Christine Kreuder Johnson, University of California, Davis, CA USA | Adult | blood | 2008 | yes |
| **GECA16** | CA | Terra Kelly, Christine Kreuder Johnson, University of California, Davis, CA USA | Adult | blood | 2008 | yes |
| **GECA18** | CA | Terra Kelly, Christine Kreuder Johnson, University of California, Davis, CA USA | Subadult | blood | 2008 | yes |
| **GECA19** | CA | Terra Kelly, Christine Kreuder Johnson, University of California, Davis, CA USA | Subadult | blood | 2008 | yes |
| **GECA20** | CA | Terra Kelly, Christine Kreuder Johnson, University of California, Davis, CA USA | Hatch Year^4^ | blood | 2008 | yes |
| **GECA21** | CA | Terra Kelly, Christine Kreuder Johnson, University of California, Davis, CA USA | Adult | blood | 2008 | yes |
| **GECA22** | CA | Terra Kelly, Christine Kreuder Johnson, University of California, Davis, CA USA | Adult | blood | 2008 | yes |
| **GECA23** | CA | Terra Kelly, Christine Kreuder Johnson, University of California, Davis, CA USA | Subadult | blood | 2009 | yes |
| **GECA24** | CA | Terra Kelly, Christine Kreuder Johnson, University of California, Davis, CA USA | Adult | blood | 2009 | yes |
| **GECA25** | CA | Terra Kelly, Christine Kreuder Johnson, University of California, Davis, CA USA | Adult | blood | 2009 | yes |
| **GECA26** | CA | Terra Kelly, Christine Kreuder Johnson, University of California, Davis, CA USA | Adult | blood | 2009 | yes |
| **GEID01** | ID | Tim and Erica Craig, Aquila Environmental, Fairbanks, AK USA | Subadult | blood | 1994 | yes |
| **GEID02** | ID | Tim and Erica Craig, Aquila Environmental, Fairbanks, AK USA | Adult | blood | 1991 | yes |
| **GEID03** | ID | Tim and Erica Craig, Aquila Environmental, Fairbanks, AK USA | Adult | blood | 1995 | yes |
| **GEID04** | ID | Tim and Erica Craig, Aquila Environmental, Fairbanks, AK USA | Adult^2^ | blood | 1996 | yes |
| **GEID05** | ID | Tim and Erica Craig, Aquila Environmental, Fairbanks, AK USA | Adult | blood | 1991 | yes |
| **GEID06** | ID | Tim and Erica Craig, Aquila Environmental, Fairbanks, AK USA | Adult | blood | 1994 | yes |
| **GEID07** | ID | Tim and Erica Craig, Aquila Environmental, Fairbanks, AK USA | Adult | blood | 1991 | yes |
| **ID19893** | ID | Tim and Erica Craig, Aquila Environmental, Fairbanks, AK USA | Juvenile^2^ | blood | 1997 | yes |
| **ID35801** | ID | Tim and Erica Craig, Aquila Environmental, Fairbanks, AK USA | Adult | blood | 1993 | yes |
| **ID35806** | ID | Tim and Erica Craig, Aquila Environmental, Fairbanks, AK USA | Juvenile^2^ | blood | 1992 | yes |
| **ID37940** | ID | Tim and Erica Craig, Aquila Environmental, Fairbanks, AK USA | Subadult | blood | 1998 | yes |
| **GEID11** | ID | James McKinley, Raptor Research Center, Boise State University, Boise, ID USA | Juvenile^2^ | blood | 2011 | yes |
| **GEID12** | ID | James McKinley, Raptor Research Center, Boise State University, Boise, ID USA | Juvenile^2^ | blood | 2011 | yes |
| **GEID13** | ID | James McKinley, Raptor Research Center, Boise State University, Boise, ID USA | Adult^3^ | feather | 2011 | yes |
| **GEID14** | ID | James McKinley, Raptor Research Center, Boise State University, Boise, ID USA | Juvenile^2^ | blood | 2011 | yes |
| **GEID15** | ID | James McKinley, Raptor Research Center, Boise State University, Boise, ID USA | Juvenile^2^ | blood | 2011 | yes |
| **GEOR01** | OR | James McKinley, Raptor Research Center, Boise State University, Boise, ID USA | Unknown | feather | 2011 | yes |
| **GEOR02** | OR | James McKinley, Raptor Research Center, Boise State University, Boise, ID USA | Juvenile^2^ | feather | 2011 | yes |
| **GEOR03** | OR | James McKinley, Raptor Research Center, Boise State University, Boise, ID USA | Juvenile^2^ | feather | 2011 | yes |
| **GEOR04** | OR | James McKinley, Raptor Research Center, Boise State University, Boise, ID USA | Juvenile^2^ | feather | 2011 | yes |
| **GEOR06** | OR | James McKinley, Raptor Research Center, Boise State University, Boise, ID USA | Adult | feather | 2011 | yes |
| **ID18887** | ID | Tim and Erica Craig, Aquila Environmental, Fairbanks, AK USA | Subadult | blood | 1998 | Winter |
| **ID18890** | ID | Tim and Erica Craig, Aquila Environmental, Fairbanks, AK USA | Adult | blood | 1998 | Winter |
| **ID18891** | ID | Tim and Erica Craig, Aquila Environmental, Fairbanks, AK USA | Adult | blood | 1998 | Winter |
| **ID19854** | ID | Tim and Erica Craig, Aquila Environmental, Fairbanks, AK USA | Adult | blood | 1996 | Winter |
| **ID35853** | ID | Tim and Erica Craig, Aquila Environmental, Fairbanks, AK USA | Adult | blood | 1993 | Winter |
| **ID35855** | ID | Tim and Erica Craig, Aquila Environmental, Fairbanks, AK USA | Subadult | blood | 1993 | Winter |
| **ID35871** | ID | Tim and Erica Craig, Aquila Environmental, Fairbanks, AK USA | Adult | blood | 1994 | Winter |
| **ID37946** | ID | Tim and Erica Craig, Aquila Environmental, Fairbanks, AK USA | Adult | blood | 1996 | Winter |
| **ID37930** | ID | Tim and Erica Craig, Aquila Environmental, Fairbanks, AK USA | Adult | blood | 1995 | Winter |
| **GEAK01** | AK | Travis Booms, Alaska Fish and Game, Fairbanks, AK USA | Juvenile^2^ | blood | 2006 | yes |
| **GEAK02** | AK | Travis Booms, Alaska Fish and Game, Fairbanks, AK USA | Juvenile^2^ | blood | 2006 | yes |
| **GEAK03** | AK | Travis Booms, Alaska Fish and Game, Fairbanks, AK USA | Juvenile^2^ | blood | 2006 | yes |
| **GEAK04** | AK | Erica Craig, Aquila Environmental, Fairbanks, AK USA | Subadult | feather | 2011 | yes |
| **GEAK05** | AK | Erica Craig, Aquila Environmental, Fairbanks, AK USA | Subadult | feather | 2011 | yes |
| **GEAK06** | AK | Erica Craig, Aquila Environmental, Fairbanks, AK USA | Unknown | fecal sample | 2011 | yes |
| **GEAK07** | AK | Tim and Erica Craig, Aquila Environmental, Fairbanks, AK USA | Adult^2^ | blood | 1994 | yes |

^1^ Idaho = ID, Oregon = OR, California = CA, Alaska = AK, Montana = MT. ^2^Banded as a nestling or fledgling. ^3^Breeding adult on territory. ^4^Eagle was sampled during the same year that it hatched.

Table B. **Size range (in base pairs), number of alleles (Na), expected and observed heterozygosity (He, Ho), probability of identity (PID, PIDsibs), and citation for each microsatellite locus that amplified in 54 golden eagles (Idaho = 24, California = 24, Alaska = 6).**

| **Locus** | **Size range** | **Na** | **He** | **Ho** | **PID** | **PIDsibs** | **Reference** |
| --- | --- | --- | --- | --- | --- | --- | --- |
| **Aa02§** | 138-146 | 4 | 0.51 | 0.52 | 0.300 | 0.573 | [2] |
| **Aa04** | 123-154 | 14 | 0.80 | 0.69 | 0.053 | 0.365 | [2] |
| **Aa11** | 246-268 | 6 | 0.75 | 0.80 | 0.092 | 0.399 | [2] |
| **Aa26** | 138-149 | 6 | 0.53 | 0.52 | 0.231 | 0.546 | [2] |
| **Aa27** | 85-97 | 5 | 0.52 | 0.46 | 0.253 | 0.557 | [2] |
| **Aa35** | 244-257 | 3 | 0.28 | 0.26 | 0.538 | 0.748 | [2] |
| **Aa36** | 84-102 | 8 | 0.63 | 0.57 | 0.164 | 0.479 | [2] |
| **Aa39** | 158-176 | 8 | 0.58 | 0.56 | 0.207 | 0.517 | [2] |
| **Aa43†** | 120-142 | 5 | 0.70 | 0.26 | 0.115 | 0.432 | [2] |
| **Aa49** | 94-108 | 8 | 0.72 | 0.74 | 0.111 | 0.422 | [2] |
| **BV13** | 180-202 | 6 | 0.59 | 0.56 | 0.188 | 0.508 | [3] |
| **Hal09** | 127-149 | 9 | 0.77 | 0.61 | 0.069 | 0.387 | [4] |
| **Hal10** | 227-246 | 7 | 0.72 | 0.70 | 0.113 | 0.421 | [4] |
| **Hal13** | 155-157 | 2 | 0.32 | 0.30 | 0.496 | 0.715 | [4] |
| **IEAAAG13** | 240-244 | 2 | 0.25 | 0.26 | 0.577 | 0.772 | [5] |
| **IEAAAG14** | 195-203 | 3 | 0.52 | 0.57 | 0.315 | 0.569 | [5] |
| **IEAAAG15** | 108-120 | 4 | 0.57 | 0.56 | 0.242 | 0.535 | [5] |
| **NVHfr142** | 173-183 | 4 | 0.35 | 0.37 | 0.422 | 0.685 | [6] |
| **Average** |  | 5.8 | 0.56 | 0.52 | 0.249 | 0.535 |  |
| **Aa12^1^** | 152-154 | 2 | - | - | - | - | [2] |
| **Aa50^1^** | 217 | 1 | - | - | - | - | [2] |
| **Age11^1^** | 245 | 1 | - | - | - | - | [7] |
| **IEAAAG05^1^** | 138-142 | 2 | 0.10 | 0.08 | - | - | [5] |
| **IEAAAG11^1^** | 187-206 | 4 | 0.55 | 0.42 | - | - | [5] |
| **NVHfr190-2^1^** | 118-132 | 4 | 0.73 | 0.08 | - | - | [6] |
| **NVHfr206^1^** | 161-163 | 2 | 0.08 | 0.08 | - | - | [6] |
| **Average** |  | 2.3 | 0.37 | 0.17 |  |  |  |

^1^Microsatellite locus that successfully amplified in a test group of 12 golden eagles but was not chosen for further analysis.

§Values for this locus were calculated from 27 individuals

†Values for this locus were calculated from 15 individuals

**References**

1. Howell SNG. Molt in North American Birds. Boston, MA USA: Houghton Mifflin Harcourt; 2010.

2. Martínez-Cruz B, David VA, Godoy JA, Negro JJ, O’Brien SJ, Johnson WE. Eighteen polymorphic microsatellite markers for the highly endangered Spanish imperial eagle (Aquila adalberti) and related species. Mol Ecol Notes. 2002;2: 323–326.

3. Gautschi B, Tenzer I, Müller JP, Schmid B. Isolation and characterization of microsatellite loci in the bearded vulture (Gypaetus barbatus) and cross-amplification in three Old World vulture species. Mol Ecol. 2000;9: 2193–2195.

4. Hailer F, Gautschi B, Helander B. Development and multiplex PCR amplification of novel microsatellite markers in the White-tailed Sea Eagle, Haliaeetus albicilla (Aves: Falconiformes, Accipitridae). Mol Ecol Notes. 2005;5: 938–940.

5. Busch JD, Katzner TE, Bragin E, Keim P. Tetranucleotide microsatellites for aquila and haliaeetus eagles. Mol Ecol Notes. 2005;5: 39–41.

6. Nesje M, Røed KH. Microsatellite DNA markers from the gyrfalcon (Falco rusticolus) and their use in other raptor species. Mol Ecol. 2000;9: 1438–1440.

7. Topinka JR, May B. Development of polymorphic microsatellite loci in the northern goshawk (Accipiter gentilis) and cross-amplification in other raptor species. Conserv Genet. 2004;5: 861–864.
